# Effects of Polymeric Crosslinker on Network Structure, Morphology, and Properties of Liquid Isoprene Rubber

**DOI:** 10.3390/polym17040551

**Published:** 2025-02-19

**Authors:** Jishnu Nirmala Suresh, Hans Liebscher, Hartmut Komber, Muhammad Tahir, Gerald Gerlach, Sven Wießner

**Affiliations:** 1 Institute of Materials Science, Faculty of Mechanical Science and Engineering, TUD Dresden University of Technology, 01062 Dresden, Germany; wiessner@ipfdd.de; 2 Department of Elastomers, Leibniz-Institut für Polymerforschung Dresden e.V., 01069 Dresden, Germany; tahir@ipfdd.de; 3 Institute of Solid-State Electronics, Faculty of Electrical and Computer Engineering, TUD Dresden University of Technology, 01062 Dresden, Germany; hans.liebscher@tu-dresden.de (H.L.); gerald.gerlach@tu-dresden.de (G.G.); 4 Center Macromolecular Structure Analysis, Leibniz-Institut für Polymerforschung Dresden e.V., 01069 Dresden, Germany; komber@ipfdd.de

**Keywords:** liquid isoprene rubber, polymeric crosslinker, dielectric elastomer actuators, actuation performance, leakage current

## Abstract

In this study, we investigated the influence of an epoxy end-capped polypropylene oxide crosslinker (epoxy-PPO) on the formation of the crosslinked network structure, the stress–strain response, and the electro-mechanical actuation performance of a maleic anhydride functionalized liquid isoprene rubber (LIR). The crosslinker amount varied from 10 (C-LIR-10) to 50 (C-LIR-50) weight parts per hundred parts (phr) of LIR. The swelling test of the cured rubbers revealed that C-LIR-20 formed the densest crosslinked network with the lowest chloroform uptake value within this series. The crosslinked rubber became stiffer in tensile response upon increasing the epoxy-PPO amount from C-LIR-10 to C-LIR-20 and then softened at higher amounts. The SEM measurements were used to relate this composition-induced softening of the rubbers to the phase morphology evolution from nanoscale homogeneity in C-LIR-10 to microscale segregations of excess crosslinkers in C-LIR-50. The use of epoxy-PPO improved the dielectric constant value of LIR; however, the leakage current through the films also increased from 25 µA DC to 320 µA DC for LIR-30 and LIR-50, respectively, during DEA operation. The electro-mechanical actuation tests with circular actuators showed that the C-LIR-10 elastomer film demonstrated a radial strain of 1.7% on activation at an electric field strength of 17.5 V/µm. At higher crosslinker amounts, the close proximity of excess epoxy-PPO molecules caused leakage current across elastomer films thus diminishing the actuation strain of otherwise relatively softer elastomers with higher dielectric constant values.

## 1. Introduction

Electroactive polymers (EAPs) are a highly versatile class of materials capable of undergoing substantial deformation in response to the electrical stimuli, making them integral to the advancements in soft robotics, artificial muscles, and adaptive systems. Among the EAPs, dielectric elastomers (DEs) have gained significant attention due to their remarkable electromechanical transduction potential, lightweight structure, and ability to generate controllable deformations under applied electric fields [1,2]. Dielectric elastomer actuators (DEAs), which leverage electrostatic forces between compliant electrodes and a dielectric medium, are especially promising, with potential applications ranging from biomedical devices to energy harvesting systems [3,4].

One of the key challenges in optimizing DEA performance is achieving a high dielectric constant without compromising mechanical integrity. Recent advancements have explored the introduction of polar functional groups into elastomers to enhance dielectric properties. Functionalized liquid isoprene rubber (LIR), modified with maleic anhydride groups, is particularly attractive for this purpose. Its ease of processing and ability to form tailored crosslinked networks make it a strong candidate for developing performance-optimized DEAs [5,6].

The introduction of polymeric crosslinkers such as epoxy end-capped polypropylene oxide (epoxy-PPO) into liquid isoprene rubber (LIR) formulations enables the formation of soft and flexible crosslinked networks. Such crosslinking networks can influence both the mechanical and dielectric characteristics and thus directly impact the actuation performance of DEA systems. The enhancement of dielectric properties through dipolar and interfacial polarization mechanisms was reported when polar moieties were introduced via crosslinkers [7,8]. Also, the concentration of crosslinkers is known to influence the density of the crosslinked network, morphological characteristics, and tensile properties of elastomeric materials [9,10,11].

This study systematically investigates the role of an epoxy-PPO crosslinker in curing LIR by varying its concentration from 10 to 50 phr. Advanced characterization techniques, including high-resolution magic angle spinning nuclear magnetic resonance (HR-MAS NMR) spectroscopy and scanning electron microscopy with energy dispersive x-ray (SEM-EDX) analysis, are employed to analyze the network structure created on curing and morphological characteristics, respectively, and then correlate their implications on tensile and actuation performance of LIR.

## 2. Materials and Methods

### 2.1. Materials

The liquid base rubber material (LIR-403) was procured from Kuraray Europe GmbH, Hattersheim, Germany. LIR-403 is a maleic anhydride functionalized liquid isoprene rubber, and its functionality is exploited in the formation of an elastomer network [12].

To crosslink the maleic anhydride grafted LIR, Struktol Polydis 3616 was employed as the liquid polymeric crosslinker. This material was purchased from Schill + Seilacher “Struktol” GmbH, Hamburg, Germany, and is composed of carboxyl-terminated nitrile-butadiene rubber (CNBR) and a low-molecular-weight epoxy end-capped polypropylene oxide (epoxy-PPO) [12].

The crosslinking reaction was catalyzed by 2,4,6-tris(dimethylaminomethyl)phenol (DMP-30), which was also purchased from Schill + Seilacher “Struktol” GmbH, Hamburg, Germany.

The formulation of rubber mixtures is provided in the Table 1. The sample designations LIR-10, LIR-20, LIR-30, LIR-40, and LIR-50 correspond to the quantity of the epoxy crosslinker in each formulation in weight parts per hundred parts rubber (phr).

### 2.2. Mixing and Molding Procedure

The initial step was the controlled mixing of LIR-403 with the epoxy crosslinker (Polydis 3616). This process was facilitated by using a SpeedMixer (DAC150 SP, Hauschild GmbH & Co. KG, Hamm, Germany). The rubber formulations reported in Table 1 were weighed into a polypropylene cup and mixed in a programmed sequence as reported in our previous work [12].

The crosslinking behavior of the liquid rubber formulations was determined by using an oscillating shear curemeter (SIS V-50, Scarabaeus Mess- und Produktionstechnik GmbH, Wetzlar, Germany) at a constant temperature of 160 °C. From these curing curves, the optimum cure times t_90_ were obtained and utilized for the compression molding of the samples by using a TP 1000 hot press (Fontijne Presses, Delft, The Netherlands) at 160 °C and applying 150 kN force.

## 3. Characterization Techniques

### 3.1. Extraction and Swelling Experiments

The cured rubber specimens were swollen in chloroform to extract out the sol fraction. The extraction experiments were each performed on a small piece of the cured material (~10 × 10 × 2 mm^3^) using chloroform (CHCl_3_, density: 1.49 g/mL) as the solvent [12]. From the weight before extraction, the weight of the swollen material after removing the last extract, and the weight of the vacuum-dried material after extraction, the solvent uptake (mL CHCl_3_/mg material) and the weight loss due to extraction (mass %) were calculated.

### 3.2. High-Resolution Magic Angle Spinning (HR-MAS) NMR Spectroscopy

The ^1^H NMR measurements were carried out using a Bruker Avance III 500 spectrometer (Bruker, Ettlingen, Germany) operating at 500.13 MHz for ^1^H. The HR-MAS NMR experiments were carried out using a special Bruker HR-MAS probe. The PTFE insert (50 μL volume) of the 4 mm ZrO_2_ rotor was filled with small pieces of the material (~2 mg). After the addition of CDCl_3_ and a 30 min swelling time, the sample was measured at υ_r_ = 4000 Hz at 30 °C. The spectra were referenced to the solvent signal (δ(^1^H) = 7.26 ppm).

### 3.3. Morphology Characterization by SEM-EDX

The cross-sections for scanning electron microscopy (SEM) were prepared using a UC6 ultramicrotome (Leica Microsystems GmbH, Wetzlar, Germany) equipped with a diamond knife, with cryo-microtomy performed at −160 °C. The SEM images were recorded using NEON40 SEM (Carl Zeiss Microscopy Deutschland GmbH, Oberkochen, Germany) operated at an acceleration voltage of 1 kV. Specimens for basic SEM imaging were left uncoated, while those used for element mapping via energy dispersive X-ray spectroscopy (EDX) in the Ultra Plus SEM (Carl Zeiss Microscopy Deutschland GmbH, Oberkochen, Germany) at 3 kV were coated with ca. 10 nm thick carbon film using a SDC 500 coater (Leica Microsystems GmbH, Wetzlar, Germany) to minimize electron beam charging.

### 3.4. Tensile Characterization

The testing of the tensile properties was carried out in accordance with the DIN 53504 standard [13]. For this purpose, the Zwick 1456, Z010 Universal Testing Machine (UTM) from ZwickRoell GmbH & Co. KG, Ulm, Germany, was utilized. Tensile tests were carried out at a uniform crosshead speed of 200 mm/min.

### 3.5. Characterization of Dielectric Properties

To handle the C-LIR-based films, a circular acrylic frame with an inner hole diameter of 150 mm was bonded with double-sided adhesive tape (Adhesive Research EL-8932EE, Adhesive Research Inc., Glen Rock, PA, USA) to the unstretched elastomer films. These samples were placed between a parallel plate capacitor setup consisting of stainless-steel electrodes, as reported in the previous studies [12,14,15]. The dielectric properties of the C-LIR-based films were analyzed using a Solartron SI 1260A frequency response analyzer (Solartron Analytical U.K., Farnborough, UK). A sinusoidal excitation voltage with a root mean square (RMS) value of 1 V was applied to the samples in the frequency range from 1 Hz to 1 MHz. The real and imaginary parts of the impedance Z* = *Z*′ + j*Z*″ were recorded with 10 points per decade. From the values of the complex impedance the real and imaginary parts of the complex permittivity *ε*_r_* = *ε*_r_′ + j*ε*_r_″ were calculated based on the following [16]:

(1) $$\varepsilon_{r}^{\prime }\left(f\right)=\frac{\left|Z^{\prime \prime }\right|K_{cell}}{2\pi f\varepsilon_{0}\left({\left(Z^{\prime }\right)}^{2}+{\left(Z^{\prime \prime }\right)}^{2}\right)}$$

(2) $$\varepsilon_{r}^{\prime \prime }\left(f\right)=\frac{\left|Z^{\prime }\right|K_{cell}}{2\pi f\varepsilon_{0}\left({\left(Z^{\prime }\right)}^{2}+{\left(Z^{\prime \prime }\right)}^{2}\right)}$$

where *f* is the variable frequency, $\varepsilon_{0}$ the permittivity in vacuum, and $K_{cell}$ the cell constant defined by the following:

(3) $$K_{cell}=\frac{d_{sample}}{A_{electrode}}$$

where $d_{sample}$ is the sample thickness and $A_{electrode}$ the area of the circular electrode.

After the impedance measurements, the C-LIR films were further processed into circular DEAs.

### 3.6. DEA Deformation Measurements

To characterize actuation performance, all the C-LIR films were radially pre-stretched by 15% and bonded to a circular acrylic frame using double-sided adhesive tape (Adhesive Research EL-8932EE, Adhesive Research Inc., Glen Rock, PA, USA). The inner diameter of the acrylic frame amounted to 100 mm.

Prior to the application of carbon paste electrodes that was prepared by mixing part-A silicone ExSil^®^50 (Gelest Inc., Morrisville, NC, USA) with 11 phr of carbon black PRINTEX^®^ XE-2B (Orion Engineered Carbons GmbH, Eschborn, Germany) using a speed mixer, the thickness of each pre-stretched film at the electrode region was precisely measured. An incremental probe (IKF 10, Feinmess Suhl GmbH, Suhl, Germany), equipped with a plate-shaped measuring insert (diameter: 4 mm) and a display unit (PU 11, Feinmess Suhl GmbH, Suhl, Germany), was used for this purpose. The average film thickness was derived by taking 20 values from each film.

Following thickness measurement, both sides of the dielectric elastomer film were coated with concentric circular electrodes of specified dimensions (diameter: 50 mm, connecting strip width: 3 mm) using adhesive stencils that were adhered to the film. The connecting strips facilitated the electrical connection to the high-voltage power supply (Peta-pico-Voltron [17]), which was essential for the actuation of the DEAs. To achieve maximum electrode displacement during DEA operation and to reduce the sensitivity to uncertainties in optical strain measurements, a ratio of electrode diameter to total film diameter of 0.5 was chosen [18].

The high-voltage power supply was configured to set voltage levels ranging from 0 to 5000 V to the DEAs. Based on the measured layer thicknesses in the electrode area, the voltage levels were calculated individually for each DEA, such that nominal electric field strengths of 5 to 17.5 V/µm in 2.5 V/µm steps were applied. The radial strain of the electrode was recorded based on video capture of the electrode area when applying the respective voltage level for 60 s.

A compact camera (Sony α6400, Sony, Tokyo, Japan) equipped with a macro lens (Sony SEL30M35, Sony, Tokyo, Japan) was utilized to observe the electrode area. This setup allowed for high-resolution capture at a speed of 100 frames per second, ensuring detailed documentation of the actuator’s deformation behavior from the non-actuated into the actuated state.

The video data were subsequently processed using MATLAB (Version 9.10 (R2021a)), which facilitated the extraction of the electrode strain. The analysis considered the increase in electrode area upon actuation, assuming an ideal circular surface expansion. This approach provided a quantitative assessment of the DEA’s performance, offering insights into the material’s suitability for applications requiring precise control over actuation and deformation.

### 3.7. Measurement of DEA Current

**Figure 1:**
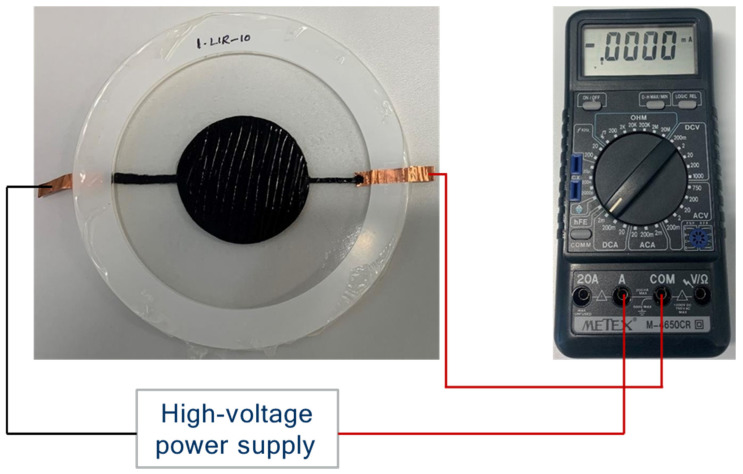
Measurement setup for DEA current during electrical activation.

The current measurement was conducted on DEAs with varying epoxy content (10 phr to 50 phr) to assess possible leakage current as the electric field strength increased. A digital multimeter (M-4650CR, Metex Corporation, Seoul, Republic of Korea) was connected in series with the DEA to measure the current in a direct current measuring range of 2 mA (Figure 1). After applying the voltage to the actuator using a high-voltage power supply, the current value was read after 60 s at each voltage level.

## 4. Results and Discussion

### 4.1. Curing Characteristics

**Figure 2:**
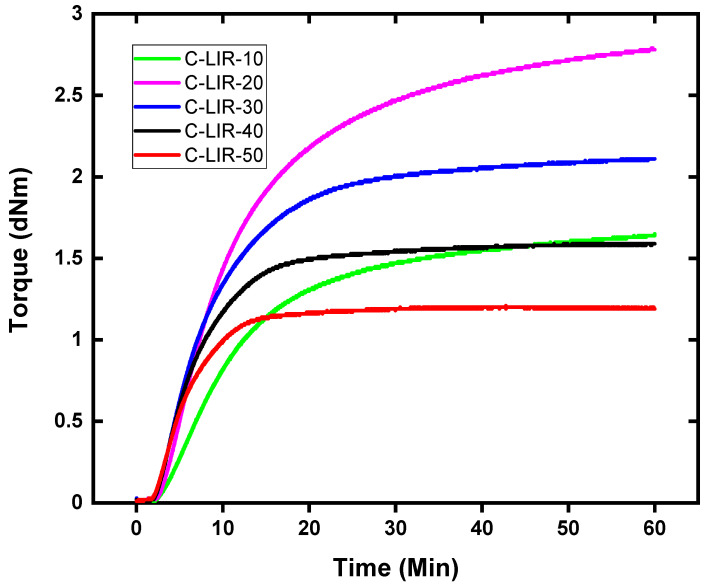
Curing profile of all LIR compounds at 160 °C.

As the curing process progressed, there was a discernible increase in torque, indicative of epoxy–anhydride esterification reactions occurring between the epoxy functionalized PPO segments and the anhydride groups present in the isoprene rubber. This reaction facilitated the formation of flexible PPO bridges that interlinked the rubber chains. The rising torque reached a plateau, indicative of the formation of a stable crosslinked network within the rubber matrix [12]. Upon extending the cure time to 60 min, the cured C-LIR samples exhibited a maximum torque value, which determined the optimal cure period required for the compression molding stage. This corresponded to t_90_, the time required to achieve 90% of the maximum torque, indicating that the material was nearly fully cured. The observed variations in torque values across different formulations highlight the influence of epoxy content on the curing dynamics and the final torque. Figure 2 clearly illustrates that C-LIR formulations with lower epoxy content, specifically C-LIR-10 to C-LIR-30, achieve a more crosslinked structure, as evidenced by their higher torque values. In the case of C-LIR-10, the limited availability of epoxy groups probably prevents the complete reaction of all anhydride groups, leading to a lesser dense network compared to C-LIR-20. The observed marching could indicate crosslinking by secondary reactions [12]. Notably, C-LIR-20 demonstrates the highest torque with a marching curve. The highest torque seems to indicate the optimal ratio of anhydride-modified rubber and epoxy crosslinker for network formation under the curing conditions used. The denser network probably has an inhibiting effect on the progress of the reaction, as can be seen from the cure curve.

The decreases in maximum torque for samples with epoxy concentrations larger than 20 phr could be attributed to two primary effects. First, crosslinking occurs when both epoxy groups of the crosslinker react with different modified isoprene rubber chains. However, if there is a lack of anhydride groups, these are quickly converted, and, for an increasing number of crosslinker molecules, the second reactive group cannot react. This leads to dangling chains but not to crosslinks, and both the crosslinking density and the torque decrease successively compared to C-LIR-20. Second, the high epoxy content may contribute to a phase separation, where epoxy-rich PPO domains form within the rubber matrix. This phase-separated morphology disrupts the uniformity of the network structure, thereby reducing the mechanical strength and consistency of the material. Together, these effects reveal the complexities of achieving optimal crosslinking density and underscore the importance of controlled epoxy content to avoid unintended microstructural changes that could compromise material performance.

### 4.2. Extraction and Swelling Tests

With the variation of the epoxy content, i.e., the crosslinker component, the question arises as to what extent this is incorporated into the network and what effect this variation has on the formation of the network. The extraction experiments aimed to determine the amount of the soluble fraction of the different cured LIR formulations. Extraction tests were carried out with chloroform as a very good solvent for both the LIR-403 and the Polydis 6313 components. While 10–12 mass % could be extracted for C-LIR-10, -20 and -30, the extractable amount increased to 15 mass % for C-LIR-40 and 20 mass % for C-LIR-50. The increase in extract for C-LIR-40 and -50 indicated that an increasing proportion of the epoxy component was not incorporated into the network during the curing process.

The ^1^H NMR spectra of the extracts provide an insight into their chemical composition. A comparison of the original reaction mixtures and the corresponding extracts show that only small amounts of LIR were present in the extracts, but increasing amounts of CNBR and epoxy-PPO originated from the Polydis 3616 component. The extractable LIR probably indicates the IR chains that were not modified with maleic anhydride groups and thus not built into the crosslinked network [12]. Such incompletely modified polymers in both LIR-403 and Polydis 3616 may be the reason for the extractable fraction of 10–12 mass % found for C-LIR-10, -20 and -30. However, the increasing amount of extractable material for C-LIR-40 and -50 is a clear indication of a saturation effect in the conversion of the epoxide groups, i.e., there were significantly more epoxide groups present than there are maleic anhydride and acid groups as counterparts. The swelling tests of the extracted samples provide a qualitative picture of the density of the network. The swelling, characterized here by the chloroform uptake in mL per gram of material, increased with decreasing network density. Similar values were found experimentally for C-LIR-10 (10.2 mL/g) and C-LIR-30 (10.3 mL/g). The chloroform uptake reached a minimum value at C-LIR-20 (8.9 mL/g), i.e., the densest network was formed. The increasing solvent uptake for C-LIR-40 (10.8 mL/g) and C-LIR-50 (11.5 mL/g) indicates a looser network. This corresponds to the lower torque values determined for both samples during the curing process compared to C-LIR-10 to -30.

### 4.3. HR-MAS NMR Spectroscopy

**Figure 3:**
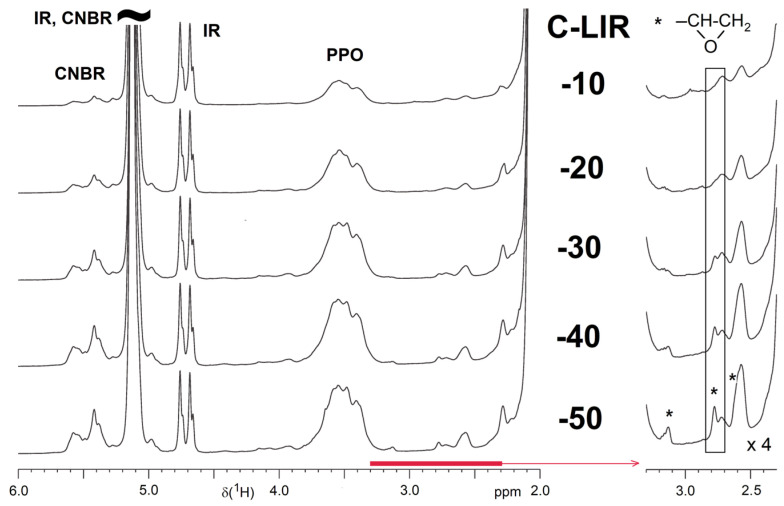
^1^H HR-MAS NMR spectra (regions) of C-LIR-x samples after extraction (swollen with CDCl_3_). The spectra were referenced to the same intensity of the signals at ca. 4.8 ppm (=CH_2_ protons of the 3,4 units of the IR). The part of the spectrum marked in red is shown on the right-hand side but enlarged to emphasize the signals of unreacted epoxide groups (marked with asterisks).

The ^1^H HR-MAS NMR measurements on the swollen C-LIR samples of different compositions after the extraction procedure provide insights into the crosslinking efficiency of the liquid isoprene rubber in the presence of different epoxy group contents. Figure 3 shows regions of the spectra of the analyzed series. The signal assignments are based on our previous study [12]. The spectra are referenced to the same intensity of the isoprene rubber (IR) signals at ca. 4.8 ppm. It is evident that the content of CNBR and PPO in the cured samples increases from C-LIR-10 to -30, but with a further increase in the amount of Polydis 3616, both components reach an upper limit in the network. This corresponds to the significantly increasing amount of non-crosslinked components in these samples observed during the extraction tests. A closer look at the conversion of epoxide groups (Figure 3) shows that signals of unreacted epoxide groups appear at C-LIR-30 and increase significantly up to C-LIR-50. After extraction, they are part of the network and represent the end groups of loose, single-side-reacted polymer chains with epoxide end groups. Such dangling chains are bound to the network but do not increase the degree of crosslinking. They probably cause the increased swelling observed in these samples. In addition, these chains and unreacted epoxy groups can have significant effects on the mechanical and dielectric properties of the material.

### 4.4. SEM-EDX Measurements

**Figure 4:**
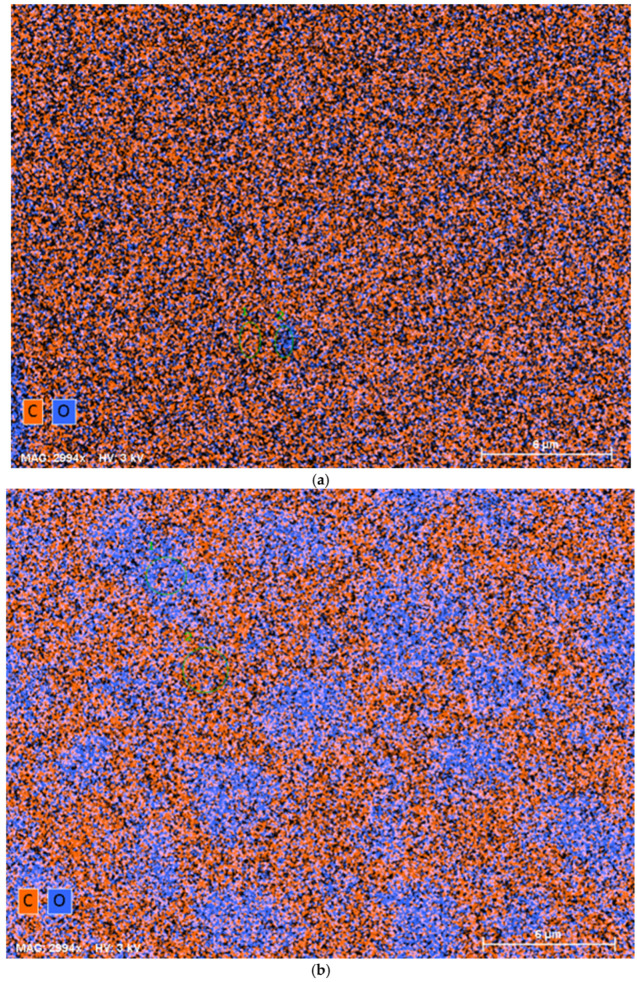
SEM-EDX images of films of (**a**) C-LIR-10 and (**b**) C-LIR-50. Blue and red represent elemental maps of oxygen and carbon, respectively.

The SEM-EDX (scanning electron microscopy with energy dispersive x-ray analysis) images of LIR cured with the lowest and highest amounts of crosslinker, i.e., C-LIR-10 and C-LIR-50, are shown in Figure 4. The differences in phase distribution and compositional contrast can clearly be seen for both elastomers.

The SEM-EDX image of C-LIR-10 elastomer (Figure 4a) displays compositional homogeneity where oxygen-rich PPO phase (blue) is very well dispersed in the carbon-rich LIR matrix (red), suggesting a successful integration of crosslinkers within the network structure. In comparison, the SEM-EDX image of C-LIR-50 (Figure 4b) shows an oxygen-rich PPO phase as micro-sized segregations embedded in a continuous crosslinked rubber network. The presence of excess epoxy-PPO was also identified in the extraction and NMR measurements. These segregations can be the un-crosslinked regions of excess crosslinker molecules that do not improve the strength of the elastomer network but may act as soft inclusions thus plasticizing the elastomer. This impact on the mechanical properties will be discussed below.

### 4.5. Tensile Test Measurements

**Figure 5:**
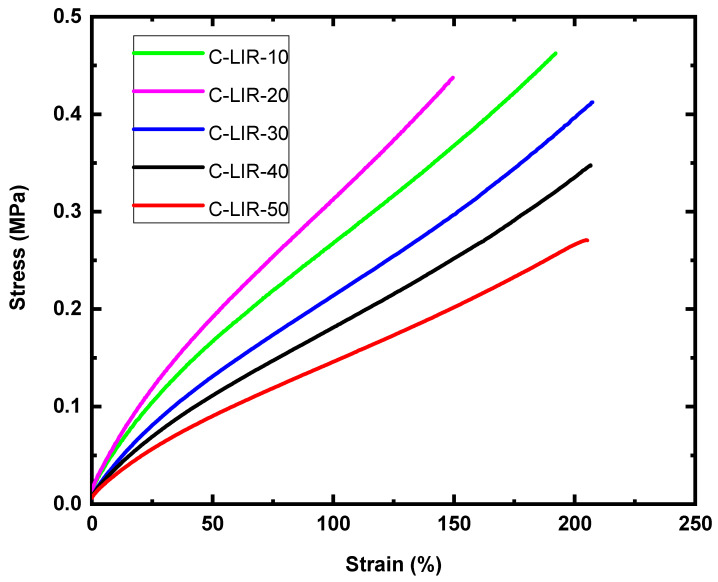
Stress–strain curves of C-LIR compounds.

The tensile test results of elastomers with varying amounts of crosslinker are plotted in Figure 5 and summarized in Table 2. All the stress–strain plots appear fairly linear, where the stress increases approximately proportionally with strain. The elastic modulus, representing the stiffness of elastomers, corresponds to the findings and conclusions discussed in Section 4.2 and Section 4.4. The modulus value is the highest for C-LIR-20, having a dense crosslinked network structure, and the lowest for C-LIR-50, having a low concentration of crosslinking points and a large excess of non-rubber fraction, as determined by solvent extraction and NMR. The stress–strain plots gradually descend from C-LIR-20 to C-LIR-50, showing higher softening with growing crosslinker addition to the rubber. The non-rubber component and phase-segregated regions within the elastomers increase with the quantity of crosslinker; as a consequence, the ultimate tensile strength drops from C-LIR-20 to C-LIR-50 in a proportional manner.

According to Maxwell’s equation [19], a low Young’s modulus of a dielectric elastomer is likely to produce large strains under an applied electric field. The results from Table 2 show that the modulus of elasticity decreases with increasing epoxy content (from C-LIR-20 to C-LIR-50). From a mechanical point of view, those formulations leading to soft elastomers could be more promising for use as dielectrics in DEAs due to the achievement of large deformations upon actuation.

### 4.6. Dielectric Properties

**Figure 6:**
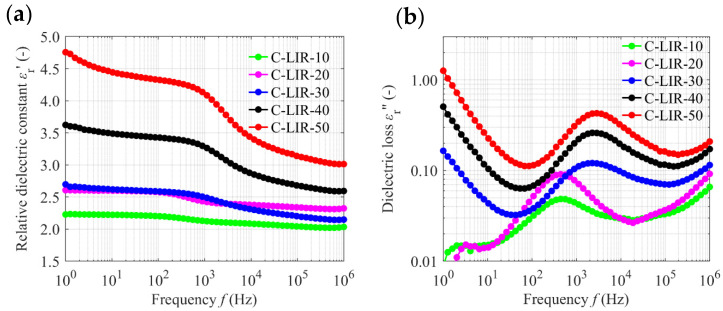
Complex permittivity spectra of C-LIR compounds. (**a**) Relative dielectric constant *ε*′ (real part), (**b**) dielectric loss *ε*″ (imaginary part).

The dielectric characterization of LIR-based elastomers is a fundamental aspect for evaluating their potential for use in DEAs. This part of the study presents the assessment of the dielectric constant and the dielectric loss across a wide frequency range, providing insights into the material’s polarization behavior and its impact on actuation performance. Figure 6 presents the complex permittivity spectra of the C-LIR samples, detailing the variations in *ε*_r_′ and *ε*_r_″ from 1 Hz to 1 MHz.

The results in Figure 6a show an increase in the dielectric constant with the addition of the epoxy crosslinker, suggesting enhanced polarizability within the C-LIR matrix. The incremental rise in dielectric constant can be attributed to the introduction of more polar species into the C-LIR network as a result of increased epoxy content. These polar entities likely introduce additional polarization mechanisms, such as dipolar and interfacial polarization, which contribute to the higher *ε*_r_′ values observed [5]. As the frequency of the sinusoidal excitation voltage increases, these polarization mechanisms may not fully respond, which is typically reflected in the frequency-dependent dielectric behavior as shown.

Plots of the dielectric loss *ε*_r_″ for all C-LIR samples are shown in Figure 6b. The imaginary parts *ε*_r_″ of C-LIR-10 and C-LIR-20 show the same behavior with only minor deviations, apart from the maximum level at 440 Hz. In the low-frequency range (1 Hz to 10 Hz), C-LIR-10 and C-LIR-20 exhibit significantly lower losses than C-LIR formulations with higher epoxy crosslinker content. Identical to *ε*_r_′, *ε*_r_″ increases with an increase in epoxy crosslinker concentration from 30 phr. In the low-frequency range (*f* < 100 Hz), an epoxy crosslinker-dependent minimum is clearly noticeable, which shifts to higher frequencies as the proportion of crosslinker increases.

The elevated dielectric constant with increased epoxy content presents a beneficial aspect for DEA performance. Higher *ε*_r_′ values imply that enhanced actuation strains can be achieved at relatively low electric field strengths, making the LIR-based DEAs more efficient in their operation. This characteristic is particularly advantageous for applications that require precise control over actuation strains without the need for high voltages. However, the dielectric loss also increases with increasing epoxy crosslinker concentration, which indicates an increase in conductivity. This would be unfavorable for use as a dielectric in DEAs [8].

### 4.7. DEA Deformation Measurements

**Figure 7:**
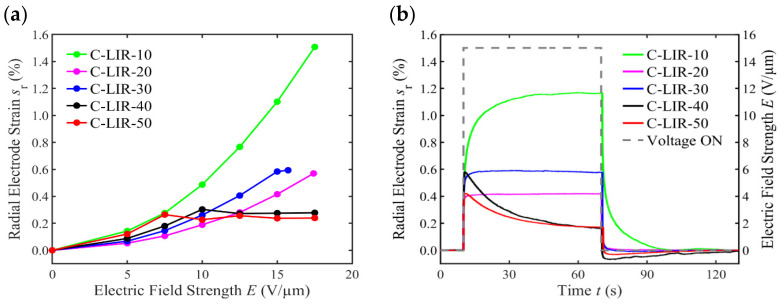
Radial electrode strain of C-LIR-based DEAs. Strain vs. (**a**) electric field and (**b**) applied electric field strength of 15 V/µm.

Figure 7a shows the radial electrode strain in relation to the electric field strength of the tested DEAs. The strain values at the respective field strengths are obtained by averaging the measured time-dependent strain signal during the time in which the electrical voltage was applied. Compared to our previous study on C-LIR-10 [12], where no pre-stretch was applied, in this case where a radial pre-stretch of 15% is used, the electro-active strain of C-LIR-10 is more than twice as high. Although the relative permittivity of C-LIR-20 is higher than that of C-LIR-10, the strains of C-LIR-20 are lower for all the tested field strengths. This is due to the higher modulus of C-LIR-20 (Figure 7). Although a higher crosslinker concentration is present in C-LIR-30 compared to C-LIR-20, the strain for C-LIR-30 is higher than that of C-LIR-20. This is an intuitive result, based on the observation that the Young’s modulus of C-LIR-30 is lower than that of C-LIR-20, and that the relative permittivity of both formulations is approximately balanced (Figure 7). However, with C-LIR-30, no further increase in strain is detected at field strengths above 15 V/µm. This behavior also persists for C-LIR-40 and C-LIR-50 with further increases in the crosslinker concentration, shifting the threshold of strain limitation to lower electric field strengths. This result indicates a current flow through the dielectric in the region of the two opposing electrodes. Due to the current flow, the charge separation cannot be maintained and the electrostatic force between the electrodes is reduced, leading to no further increase in radial electrode strain.

Figure 7b reports the time-dependent electrode strain of each DEA for a nominal electric field strength of 15 V/µm. Contradicting our previous study on C-LIR-10 [12], where almost ideal elastic behavior was observed for the voltage-induced deformation and passive recovery of the electrode, here, it takes approx. 30 s until the electrode strain reaches a constant value for C-LIR-10. Also, after switching off the electrical voltage, it takes approx. 30 s for the electrode to return to its original shape. The reason for this inconsistency could lie in the manual manufacturing process, which might have introduced irregularities in the paste-like electrode’s thickness after application. These irregularities can affect the elastic properties of the DEA, significantly impacting the time-dependent deformation behavior. The C-LIR-20 and C-LIR-30 elastomers show a rectangular-shaped square wave response in electrode strain, which indicates linear elastic material behavior. C-LIR-40 and C-LIR-50 show a sudden increase in electrode strain when the electrical voltage is switched on. However, this is followed by an immediate exponential decrease, where the final value is not reached within the time when the voltage is switched on. When the electrical voltage is switched off, the strain of C-LIR-40 and C-LIR-50 DEAs is immediately negative, which means that the electrode is in a compressed state in the radial direction. This compression is reduced over approx. 60 s until the electrode returns to its original size. This finding indicates that, as a result of the electric field, the alignment of the dipoles leads to an alignment of the polymer structure in which the polymer chains move closer together. This process leads to a small shrinkage of the recorded circular electrode area over time. When the electric field is switched off, it takes a certain amount of time for the polymer chains to return to their original position, while the electrode area increases back to its initial size. Hence, when the C-LIR-30, C-LIR-40, and C-LIR-50 DEAs are electrically activated, a superposition of current flow and the contraction of the polymer chains due to dipole alignment could lead to a decrease in electrode strain.

### 4.8. Measurement of DEA Current

**Figure 8:**
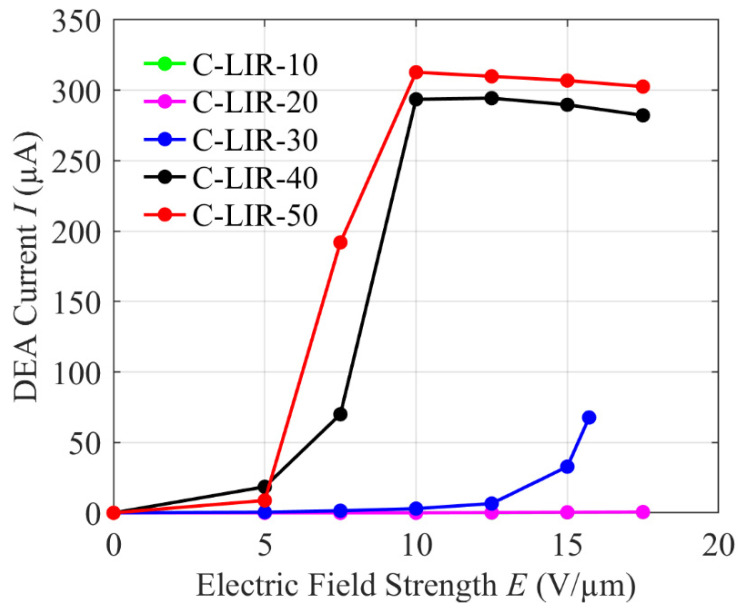
Electric field strength vs. DEA current of C-LIR-based DEAs.

Figure 8 shows that the measured values of the current after the activation voltage has been applied for 60 s. While the C-LIR-10 and C-LIR-20 DEAs exhibit no measurable current when actuated in the entire field strength range, a low current of 3 µA is detected in the C-LIR-30 DEA at a field strength of 10 V/µm. Upon further increasing the field strength to 16 V/µm, this current flow increases to a value of 77 µA. Note that the C-LIR-10 curve is not visible in the figure as it overlaps with the C-LIR-20 curve.

For DEAs from C-LIR-40 and C-LIR-50, currents of 19 µA and 8 µA, respectively, are already measured from the lowest tested field strength of 5 V/µm. When the field strength is doubled to 10 V/µm, the current rises to significantly higher values of 294 µA for C-LIR-40 and 313 µA for C-LIR-50. At field strengths above 10 V/µm, the current flow does not increase further for both C-LIR-40 and C-LIR-50 DEAs. The current assumes a plateau-like curve, which decreases slightly when higher field strengths are applied.

Overall, in the tested field strength range from 5 V/µm to 17.5 V/µm, C-LIR-10 and C-LIR-20 show no leakage current effects, which indicates good insulation properties and material integrity. In contrast, the leakage current becomes significant at higher epoxy concentrations of C-LIR-30. Particularly, C-LIR-40 and C-LIR-50 show a measurable current flow even at low field strengths (5 V/µm), which increases sharply up to a field strength of 10 V/µm and finally saturates. This could be attributed to the polar nature of the epoxy groups, which facilitates charge movement and obviously increases electrical conductivity, also increasing the risk of leakage current. Additionally, the non-reacted epoxidized PPO may form a separate electron-transporting microphase within the crosslinked LIR matrix, further contributing to the increase in leakage current by creating localized pathways for charge movement [20,21,22].

## 5. Conclusions

This study reveals that the quantity of epoxy-PPO crosslinker significantly impacts the formation of three-dimensional network structures, as well as the tensile and electro-mechanical actuation performance of an isoprene elastomer. The elastomer C-LIR-20 forms the densest network, offering both the highest torque development during the curing process and the optimal Young’s modulus (stiffness) value. Very high crosslinker-to-rubber ratios, for example, in C-LIR-50 leads to a heterogenous morphology with crosslinker-segregated regions that behave as soft inclusions that influence the strength of the elastomer network. The elastomer C-LIR-10 exhibits compositional homogeneity, as revealed by SEM-EDX, which indicates the successful integration of crosslinker molecules into the network structure. For DEA applications, C-LIR-10 shows quite a promising actuation performance compared to the higher crosslinker formulations. Although the higher crosslinker levels in C-LIR-30 to C-LIR-50 elastomers demonstrate higher values of dielectric constant and lower elastic modulus values, the leakage current increases through the dielectric elastomer film, reducing the electrostatic actuation strain.

## Figures and Tables

**Table 1:** Formulation of the rubber compounds in phr.

Components	Compounds				
	LIR-10	LIR-20	LIR-30	LIR-40	LIR-50
LIR-403	100	100	100	100	100
Struktol Polydis 3616	10	20	30	40	50

DMP-30 was used at a concentration of 1 phr in all the formulations.

**Table 2:** Young’s modulus of C-LIR compounds.

Calculated Young’s Modulus (MPa) at 15% Strain					
**Samples**	C-LIR-10	C-LIR-20	C-LIR-30	C-LIR-40	C-LIR-50
**Modulus (MPa)**	0.33	0.38	0.26	0.22	0.18

## Data Availability

Data are contained within the article.

## Abbreviations

The following abbreviations are used in this manuscript:

C-LIR	Crosslinked Liquid Isoprene Rubber
DEA	Dielectric Elastomer Actuator
DE	Dielectric Elastomer
EAP	Electroactive Polymer
PPO	Polypropylene Oxide
HR-MAS NMR	High-Resolution Magic Angle Spinning Nuclear Magnetic Resonance
SEM-EDX	Scanning Electron Microscopy–Energy Dispersive X-ray
LIR	Liquid Isoprene Rubber
phr	Parts per Hundred Rubber
CNBR	Carboxylated Nitrile-Butadiene Rubber
DMP	2,4,6-Tris(dimethylaminomethyl)phenol
CHCl_3_	Chloroform
UTM	Universal Testing Machine
RMS	Root Mean Square
NMR	Nuclear Magnetic Resonance
DIN	Deutsches Institut für Normung
IR	Isoprene Rubber
PU	Polyurethane
FEI	Field Emission Imaging
ZrO_2_	Zirconium Dioxide
PTFE	Polytetrafluoroethylene
